# Editorial: Applications of novel gene editing tools and approaches in plants

**DOI:** 10.3389/fgeed.2024.1404959

**Published:** 2024-04-08

**Authors:** Cheng Yuan, Tong Zhang, Changjun Huang

**Affiliations:** ^1^ Yunnan Academy of Tobacco Agricultural Sciences, Key Laboratory of Tobacco Biotechnological Breeding, National Tobacco Genetic Engineering Research Center, Kunming, China; ^2^ Guangdong Province Key Laboratory of Microbial Signals and Disease Control, College of Plant Protection, South China Agricultural University, Guangzhou, China

**Keywords:** genome editing tools, trait improvement, gene function, gene knockout, genetic transformation

The integration of gene editing technologies, particularly CRISPR/Cas systems, into plant biotechnology marks a groundbreaking advancement in the manipulation and understanding of plant genomes (Knott and Doudna, 2018; Manghwar et al., 2019). In this Research Topic, experts from nine groups have illustrated the rapid progress in the field, showcasing how these novel tools are revolutionizing the way we enhance crop resilience, yield, and quality traits through precise genetic modifications.

The discussion initiated by Ramesh et al. underscores the transformative potential of CRISPR/Cas9 in palm breeding and addresses the critical needs for sustainability and productivity in palm cultivation. Further expanding the scope of CRISPR/Cas9’s impact, Yao et al.'s comprehensive review explores its applications in soybean molecular breeding. The authors detail how gene editing is being utilized to confer resistance to abiotic stresses and diseases, improve seed quality, and manipulate plant architecture for better yields. Vu et al.'s review delves into the utilization of CRISPR-Cas systems for enhancing plant tolerance to stress. Focusing on the unfolded protein response (UPR) as a mechanism to combat stress-induced damage (Liu et al., 2022), the review highlights how targeting specific UPR-related genes can lead to the development of crops with improved stress tolerance. The original research from Bhowmik et al. focuses on modifying the lipoxygenase (LOX) gene in yellow peas using CRISPR/Cas9, aiming to enhance sensory qualities by reducing off-flavors. This targeted genetic modification exemplifies the nuanced applications of gene editing in improving the nutritional and sensory attributes of food crops, offering new possibilities for food application without the need for post-harvest processing.

Optimizing Agrobacterium-mediated genetic transformation is crucial for CRISPR genome editing in plants, as it significantly enhances the efficiency and reliability of gene delivery, facilitating precise genetic alterations and advancing plant biotechnology (Chen et al., 2022). Ma et al. presents a groundbreaking *Agrobacterium rhizogenes*-mediated genetic transformation technology for citrus. This method streamlines the genetic modification process, demonstrating its efficacy across various citrus genotypes and enabling efficient study of genes previously challenging to analyze. While Yuan et al. develops a highly efficient CRISPR/Cas9 system delivered by Agrobacterium for genome editing in wild tobacco (*Nicotiana alata*), overcoming the challenge of lacking an efficient genetic transformation and genome editing system in this species. This study not only advances genetic research in tobacco, but also sets a precedent for applying genome editing technologies to other species lacking efficient genetic transformation and editing systems.

Transformation in soybean is challenging due to its complex genetic structure and low efficiency in traditional methods, making protoplast transfection crucial for enabling direct gene editing within cells (Xu et al., 2022). The use of ribonucleoprotein (RNP) complexes in this process offers a significant advantage by enhancing precision, reducing off-target effects, and facilitating transient expression without integrating foreign DNA into the genome. Subburaj and Agapito-Tenfen develops a CRISPR/Cas9 RNP-based genome editing method for soybean protoplasts using electro-transfection, enhancing targeted mutagenesis in soybean by bypassing the need for PEG-mediated transfection methods. This innovation represents a significant leap in soybean genetic engineering, providing a more precise and efficient approach to improving crop traits and conducting functional genomics studies.

Optimizing and discovering promoters is pivotal for plant transformation and gene editing as it directly influences gene expression levels and specificity, thereby ensuring the effectiveness and precision of genetic modifications in plants. Ye et al. identifies and applies novel promoters from Chinese fir to enhance the efficiency of CRISPR/Cas-mediated genome editing in plant protoplasts. This strategy not only advances genetic engineering in forestry but also showcases the potential for these promoters to facilitate genetic improvements across a broader range of plant species.

Using CRISPR for pathogen detection is important because it offers a highly specific, sensitive, and rapid method to identify pathogens, significantly improving disease management and prevention strategies in agriculture. Wang et al.'s research introduces a novel CRISPR/Cas12a-based visual nucleic acid detection system for identifying plant viruses, such as *sorghum mosaic virus* (SrMV) and *rice stripe mosaic virus* (RSMV), in the field. This rapid and sensitive method offers a practical tool for early detection and management of viral infections, thereby mitigating potential impacts on crop production.

Collectively, these articles underscore the profound impact of CRISPR/Cas9 and related technologies on plant biotechnology. From enhancing crop resilience and yield to innovating disease diagnostics and genetic studies, gene editing tools are paving the way for the development of next-generation crops with improved performance and sustainability (Rodriguez-Leal et al., 2017; Gao, 2021). As these technologies continue to evolve, their application in addressing the complex challenges of modern agriculture holds great promise for advancing global food security and environmental sustainability.
